# Hormone and HER2-receptor status in breast cancer: determination using sonographically guided core needle biopsy and correlation with excision specimen—a German single institution diagnostic accuracy study

**DOI:** 10.1007/s00404-024-07920-5

**Published:** 2025-02-06

**Authors:** Maximilian Pruss, Jan-Philipp Cieslik, Janet Török, Jerome Dobrowolski, Melissa Neubacher, Martina Helbig, Verena Friebe, Lena Häberle, Natalia Krawczyk, Felix Borgmeier, Tanja Fehm, Frederic Dietzel, Svjetlana Mohrmann

**Affiliations:** 1 Department of Obstetrics and Gynecology, Medical Faculty, Heinrich Heine University Duesseldorf, Moorenstraße 5, 40225 Düsseldorf, Germany; 2 Med 360°, Breast Imaging Center of Radiology, Luegallee 52, 40545 Düsseldorf, Germany; 3 Institute of Pathology, Medical Faculty, University Hospital Duesseldorf, Heinrich Heine University, 40204 Düsseldorf, Germany; 4 MVZ Amedes for Prenatal-Medicine und Genetic GmbH, 40210 Düsseldorf, Germany; 5 Department of Diagnostic and Interventional Radiology, Medical Faculty, University Duesseldorf, 40225 Duesseldorf, Germany

**Keywords:** Breast cancer, Core needle biopsy, Hormone receptor status, HER2-receptor status, Concordance rate

## Abstract

**Background:**

Sonographically guided core needle biopsy (CNB) is a well-established tool for diagnosing breast lesions. Preoperative estrogen receptor (ER), progesterone receptor (PR), and HER2-receptor status are essential for a personalized treatment approach.

**Objectives:**

We evaluated the concordance of the hormone- and HER2-receptor status between the CNB and the surgical specimen to determine the accuracy of the CNB as a diagnostic method.

**Design:**

This is a non-interventional retrospective study analyzing breast cancer patients treated at the breast care center of the University Medical Center Duesseldorf between January 2002 and December 2005.

**Methods:**

Patients with paired CNB and surgical specimens and a diagnosis of invasive breast cancer were included. ER, PR, and HER2 status were determined by immunohistochemistry (IHC). Patients with IHC 2+ results were further examined by fluorescence in situ hybridization (FISH). Concordance of receptor status was calculated using specificity, sensitivity, and negative and positive predictive values.

**Results:**

We found a very good agreement between CNB and surgical specimens regarding receptor status. A total of 248 patients were analyzed. Concordance rates in cases of primary surgery for ER, PR, and HER2 were 92.9%, 92.9%, and 93%, respectively. In cases of neoadjuvant chemotherapy, the concordance rates for ER, PR, and HER2 were 100%, 87.5%, and 96%, respectively.

**Conclusion:**

CNB demonstrated high diagnostic accuracy compared with surgical specimens regarding ER, PR, and HER2-receptor status. Our findings support the recommendation to use sonographically guided CNB as the initial diagnostic method for guiding tailored treatment plans.

## What does this study add to the clinical work

**Table:** 

This study supports the current body of evidence that a sonographically guided core needle biopsy is an accurate diagnostic method to evaluate the receptor status of newly diagnosed breast cancer.	

## Introduction

Breast cancer remains a formidable health challenge, accounting for a significant proportion of cancer diagnoses globally [1]. Within the intricate landscape of breast cancer, the identification and characterization of tumor biomarkers have emerged as crucial determinants guiding therapeutic decisions and prognostic evaluations. Among these biomarkers, hormone receptor status—including estrogen receptor (ER) and progesterone receptor (PR) expression—and human epidermal growth factor receptor 2 (HER2) play pivotal roles in influencing treatment modalities and patient outcomes [2, 3].

Minimally invasive techniques, especially sonographically guided core needle biopsy (CNB), enable precise molecular profiling, allowing clinicians to tailor treatment strategies based on a detailed understanding of tumor biology [4–6].

Despite the potential advantages of CNB, questions persist regarding its concordance with excision specimens, particularly in the context of tumor heterogeneity—a phenomenon wherein distinct molecular subtypes coexist within the same tumor mass [7, 8]. Tumor heterogeneity poses a significant challenge, increasing the risk of sampling errors and misrepresenting the tumor’s biology. Thus, examining the correlation between CNB findings and excision specimens is essential for enhancing diagnostic accuracy and optimizing treatment decisions.

This study aims to delineate the strengths and limitations of sonographically guided CNB in breast cancer diagnostics. By comparing CNB findings with excision specimens, it aims to inform clinical practice, improve patient care, and contribute to advancements in breast cancer research.

## Methods

This is a retrospective, non-interventional study. The study was reviewed and assessed by the ethics committee of the Heinrich Heine University in Düsseldorf. The study number is 4511.

### Patient population

**Fig. 1:**
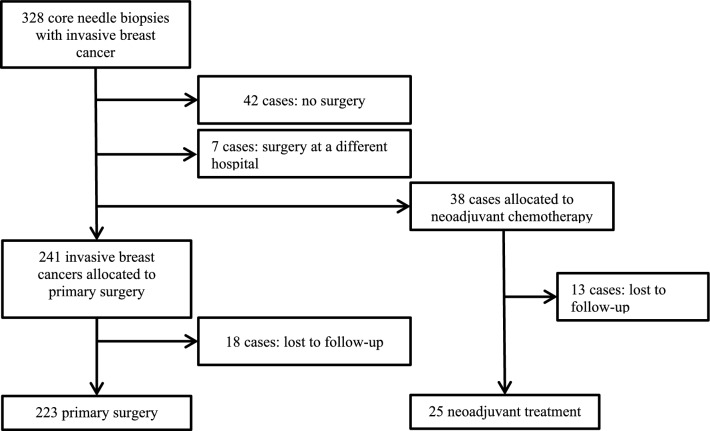
Flow chart of patients included into the study cohort

This is a non-interventional retrospective study analyzing breast cancer patients between January 2002 until December 2005 with paired CNB and surgical specimen samples that were treated in the breast care center of the University Medical Center Duesseldorf. A total of 836 CNB’s were performed for suspected malignancy and 328 (39%) had the histopathological result of invasive breast cancer. Of 328 sonographically guided CNB, 223 (68%) patients underwent primary surgery and 25 (8%) patients underwent NAC (neoadjuvant chemotherapy) prior to operation. Included were all 248 patients with a diagnosis of invasive breast cancer, that were treated at our breast care center, that were able to be followed up (Fig. 1). All patients with a benign or inconclusive histologic result, as well as patients that were lost to follow-up or chose not to be treated/treated at a different facility, were excluded.

Data included age of patient, number of CNB specimens obtained, length of the CNB specimens, tumor size, number of tumor foci, TNM stage, operation type, NAC history, ER, PR, HER2, nuclear grade. The receptor status of the surgical specimen was defined as the true receptor status.

### ER, PR and HER2 evaluation

The hormone and HER2-receptor analysis on the surgical specimen was performed on a representative tumor block. Due to the fact, that immunohistochemistry is not routinely repeated on the excision specimen when the primary diagnosis has already been established via CNB, in each case the missing receptor analysis was performed.

The immunhistochemical and FISH analyses were performed by at least two experienced gynecopathologists/breast pathologists. In unclear cases, the opinion of a senior consultant was sought.

All standardized working steps for immunohistochemistry analysis were performed according to the Labeled-Streptavidin–Biotin-Method (LSAB-Method).

We used monoclonal antibodies for nuclear staining of ER (SP1 clone, DCS Innovative Diagnostic-Systems, Hamburg, Germany) and PR (SP2 clone, DCS Innovative Diagnostic-Systems, Hamburg, Germany). Hormone receptor positive breast cancers were defined as ≥1% immunoreactive tumor cell nuclei for ER and/or PR.

HER2 status (HercepTest™ mAb pharmDx Dako Omnis, clone c-erbB-2, Agilent, Santa Clara, CA, USA) was first determined by IHC and scored as 0–3+ according to ASCO/CAP (American Society of Clinical Oncology/College of American Pathologists) guideline [1].

Immunohistochemistry is recommended as the primary examination method for the detection of HER2-positive and HER2-negative breast carcinomas. The intensity and extent of staining are expressed on a scale from 0 to 3, graded as HER2-negative breast cancer: score 0 and score 1, HER2-unclear breast cancer: score 2+ and HER2-positive breast cancer: score 3+. In the case of an unclear HER2 status (score 2+) in immunohistochemistry fluorescence in situ hybridization (FISH) is added to detect the HER2 genes that a breast carcinoma has formed. For the purpose of this study, all samples were analyzed using FISH, even when clinically not necessary (IHC 0/3, IHC 1/3), only in cases of insufficient tumoral material a FISH analysis was not performed. This results in the high number of performed FISH analyses. HER2-positive breast cancer was defined either due to HER2 expression in immunohistochemistry (score 3+) or FISH was considered as amplified (Ratio HER2 gene signals to chromosome 17 signals ≥2.0). Due to the retrospective character of the study, the today routinely used classification of HER2-low and HER2-ultralow tumors was omitted, because of the uncommon use during data acquisition.

### Statistical analysis

Concordance analysis of receptor status and molecular subtypes was performed on CNB and surgical samples. To assess the standard distribution of correlating and discrepant receptor status of analyzed tumors the Shapiro–Wilk test with a significance level of <0.05 was used.

Expression-status of ER, PR, and HER2 were categorized as described above in receptor-positive and receptor-negative. Concordance of the results from CNB and those from surgical excision was calculated using sensitivity and specificity as well as the negative and positive predictive values (NPV, PPV).

For the comparison of breast carcinomas with a concordant receptor and breast carcinomas with a discrepant receptor regarding CNB and EB (excision biopsy) the *χ*^2^ test or Fisher’s exact test, as well as the exact significance of the Mann–Whitney *U* test was calculated for two independent samples.

The data collected was analyzed using the statistical program IBM Statistical Package for the Social Sciences (SPSS Statistics) Version 28.0.1.1 for Microsoft Windows. To adjust for multiple testing *p* values were corrected via the false discovery rate algorithm where appropriate. Adjusted *p* values are referred to as *q*-values. Further, we utilized R (version 4.2.2) to perform multinomial linear regression analysis, estimates were reported as *β* values.

## Results

There were 248 breast cancer patients eligible for this study. Median age of the patients was 63 (24–90) years. Most patients received primary surgery (*n* = 223—89.9%), whereas a much smaller number of patients was treated neoadjuvantly (*n* = 25—10.1%). Not for every patient the receptor status of the CNB was known to compare to the surgical specimen. In the group of patients treated with primary surgery, the hormone receptor status in CNB was known for 198 out of 223 patients (88.7%). Regarding the HER2 Status, 220 out of 223 (98.7%) could be correlated by IHC results and 215 out of 223 (96.4%) could be correlated by results of FISH analysis. In the group of patients treated neoadjuvantly none of the patients achieved a residual cancer burden (RCB) 0. Therefore, for 15 patients, the CNB could be correlated with the surgical specimen regarding the ER receptor (60.0%) and for 16 patients regarding the PR receptor (64.0%). With regard to the HER2 status, in all 25 patients (100%) the CNB result could be correlated with the surgical specimen for IHC and FISH analysis results due to the presence of residual cancer cells.

### Correlation of CNB with surgical specimen for hormone receptor status in cases of primary surgery

Evaluation of ER expression (Tables 1, 4) on CNB samples had a 92.9% concordance rate with ER results on the surgical specimen (184/198 tumors) with ER positive in 75.3% (149/198 tumors) and ER negative tumors in 17.7% (35/198 tumors) of cases, revealing a good agreement. A discrepancy was present in 14 cases which correlates with 7.1%. The CNB result for the ER status was false negative in 5.1% of cases (8/157 ER positive tumors), therefore the CNB had a sensitivity of 94.9% with a PPV of 96.1%. Furthermore, the CNB had a 14.6% false positive rate (6/41 ER negative tumors) which translates into a specificity of 85.4% with a NPV of 81.4%.

**Table 1:** Analysis of the concordance and discrepancies between CNB and EB for estrogen receptor status (ER-Status) in cases of primary surgery

Characteristics	ER-status (total of 198 patients with invasive disease)		
	Discrepant	Concordant	*q*-value (*p* value)
CNB			
Cores (mean, SD)	2–3 (2.67; 0.52)	1–9 (2.95; 1.28)	0.569 (0.533)
Length/size in cm (SD)	3.71 (1.35)	3.21 (1.59)	0.313 (0.134)
Breast surgery			–
Breast-conserving surgery	9 (64.3%)	117 (63.6%)	
Mastectomy	5 (35.7%)	67 (36.4%)	
Invasive breast cancer			
Tumor size in cm (mean, SD)	2.06 (0.92)	2.37 (1.82)	0.569 (0.569)
T1	6/14 (42.9%)	87/184 (47.3%)	
T2	8/14 (57.1%)	87/184 (47.3%)	
T3	0	8/184 (4.3%)	
T4	0	2/184 (1.1%)	
Axillary lymph node status			0.569 (0.549)
N1	3/14 (21.4%)	60/184 (32.6%)	
N0	10/14 (71.4%)	116/184 (63.0%)	
Unknown	1/14 (7.1%)	8/184 (4.4%)	
Tumor foci			0.542 (0.310)
Unifocal	13/14 (92.9%)	142/184 (77.2%)	
Bifocal	1/14 (7.1%)	26/184 (14.1%)	
Multiple	0	16/184 (8.7%)	
Histological tumor type			0.245 (0.070)
Ductal/NST	13/14 (92.9%)	127/184 (69.0%)	
Lobular	0	26/184 (14.1%)	
Other	1/14 (7.1%)	31/184 (16.9%)	
Noninvasive tumor type			–
DCIS	9/9 (100%)	85/104 (81.7%)	
LCIS	0	19/104 (18.3%)	
Tumor grade			0.140 (0.020)
G1	0	21/184 (11.4%)	
G2	7/14 (50.0%)	124/184 (67.4%)	
G3	7/14 (50.0%)	38/184 (20.7%)	
Unknown	0	1/184 (0.5%)	

*ER* estrogen receptor, *CNB* core needle biopsy, *SD* standard deviation, *NST* no special type, *DCIS* ductal carcinoma in situ, *LCIS* lobular carcinoma in situ

The concordance of the PR receptor status (Tables 2, 4) was identical with 92.9% (184/198 tumors). Therefore, a discrepancy of the CNB and the surgical specimen was present in 7.1% of cases. Of those cases, 4.8% had a false positive PR result (7/145 tumors) and 13.2% had a false negative PR result (7/53 tumors). Those results translate into a sensitivity and PPV of 95.2%, as well as a specificity and NPV of 86.8%.

**Table 2:** Analysis of the concordance and discrepancies between CNB and EB for progesterone receptor status (PR-Status) in cases of primary surgery

Characteristics	PR-status (total of 198 Patients with invasive disease)		
	Discrepant	Concordant	*q*-value (*p* value)
CNB			
Cores (mean, SD)	3–4 (3.33; 0.58)	1–9 (2.92; 1.26)	0.656 (0.375)
Length/size in cm (SD)	3.40 (1.58)	3.23 (1.58)	0.777 (0.708)
Breast surgery			–
Breast-conserving surgery	11/14 (78.6%)	115/184 (62.5%)	
Mastectomy	3/14 (21.4%)	69/184 (37.5%)	
Invasive breast cancer			
Tumor size in cm (mean, SD)	1.96 (1.89)	2.38 (1.81)	0.343 (0.147)
T1	9/14 (64.3%)	84/184 (45.7%)	
T2	4/14 (28.6%)	91/184 (49.5%)	
T3	1/14 (7.1%)	7/184 (3.8%)	
T4	0	2/184 (1.1%)	
Axillary lymph node status			0.777 (0.777)
N1	4/14 (28.6%)	59/184 (32.1%)	
N0	10/14 (71.4%)	116/184 (63.0%)	
Unknown	0	9/184 (4.9%)	
Tumor foci			0.777 (0.738)
Unifocal	12/14 (85.7%)	143/184 (77.7%)	
Bifocal	2/14 (14.3%)	26/184 (14.1%)	
Multiple	0	15/184 (8.2%)	
Histological tumor type			0.343 (0.123)
Ductal/NST	7/14 (50.0%)	133/184 (72.3%)	
Lobular	2/14 (14.3%)	24/184 (13.0%)	
Other	5/14 (35.7%)	27/184 (14.7%)	
Noninvasive tumor type			–
DCIS	3/3 (100%)	91/110 (82.7%)	
LCIS	0	19/110 (17.3%)	
Tumor grade			0.014* (0.002)
G1	1/14 (7.1%)	20/184 (10.9%)	
G2	4/14 (28.6%)	127/184 (69.0%)	
G3	8/14 (57.1%)	37/184 (20.1%)	
Unknown	1/14 (7.1%)	0	

*ER* estrogen receptor, *CNB* core needle biopsy, *SD* standard deviation, *NST* no special type, *DCIS* ductal carcinoma in situ, *LCIS* lobular carcinoma in situ

*p ≤ 0.05

### Correlation of CNB with surgical specimen for HER2-status in cases of primary surgery

Evaluation of the HER2-status (Tables 3, 4) with IHC and FISH analysis resulted in a 93% concordance of CNB and surgical specimen, resulting in a discrepancy of 7% (15/215 tumors). There was a 25% false HER-2 negative rate (8/32 tumors) and a 3.8% false HER-2 positive rate (7/183 tumors). Those results correlate with a sensitivity and specificity of the CNB of 75% and 96.2% respectively, with a PPV of 77.4% and NPV of 95.7%.

**Table 3:** Analysis of the concordance and discrepancies between CNB and EB for human epidermal growth factor receptor 2 (HER2-Status) in cases of primary surgery

Characteristics	HER2-status (total of 215 patients with invasive disease)		
	Discrepant	Concordant	*q*-value (*p* value)
CNB			
Cores (mean, SD)	1–5 (3.00, 1.29)	1–9 (2.99, 1.24)	0.999 (0.912)
Length/size in cm (SD)	2.97 (1.26)	3.27 (1.58)	0.999 (0.625)
Breast surgery			–
Breast-conserving surgery	5 (33.3%)	(64.0%)	
Mastectomy	8 (53.3%)	(35.5%)	
Other	2 (13.3%)	1 (0.5%)	
Invasive breast cancer			
Tumor size in cm (mean, SD)	2.45 (1.44)	2.37 (1.75)	0.999 (0.872)
T1	6/15 (40.0%)	92/200 (46.0%)	
T2	7/15 (46.7%)	99/200 (49.5%)	
T3	1/15 (6.7%)	7/200 (3.5%)	
T4	1/15 (6.7%)	2/200 (1.0%)	
Axillary lymph node status			0.327 (0.133)
N1	7/15 (46.7%)	60/200 (30.0%)	
N0	6/15 (40.0%)	126/200 (63.3%)	
Unknown	2/15 (13.3%)	14/200 (7.0%)	
Tumor foci			0.327 (0.083)
Unifocal	9/15 (60.0%)	164/200 (82.0%)	
Bifocal	4/15 (26.7%)	23/200 (11.5%)	
Multiple	2/15 (13.3%)	13/200 (6.5%)	
Histological tumor type			0.327 (0.140)
Ductal/NST	8/15 (53.3%)	145/200 (72.5%)	
Lobular	1/15 (6.7%)	28/200 (14.0%)	
Other	6/15 (40.0%)	27/200 (13.5%)	
Noninvasive tumor type			–
DCIS	9/9 (100%)	91/111 (82.0%)	
LCIS	0	20/111 (18.0%)	
Tumor grade			0.999 (0.999)
G1	3/15 (20.0%)	17/200 (8.5%)	
G2	9/15 (60.0%)	135/200 (67.5%)	
G3	3/15 (20.0%)	47/200 (23.5%)	
Unknown	0	1/200 (0.5%)	

*ER* estrogen receptor, *CNB* core needle biopsy, *SD* standard deviation, *NST* no special type, *DCIS* ductal carcinoma in situ, *LCIS* lobular carcinoma in situ

**Table 4:** Concordance between CNB and EB for receptor status

CNB	EB		Concordance rate (%)	Sensitivity (%)	Specificity (%)	PPV (%)	NPV (%)
	Positive	Negative					
ER			92.9	94.9	85.4	96.1	81.4
Positive	149	6					
Negative	8	35					
PR			92.9	95.2	86.8	95.2	86.8
Positive	138	7					
Negative	7	46					
HER2			93.0	75.0	96.2	77.4	95.7
Positive	24	7					
Negative	8	176					

*CNB* core needle biopsy, *EB* excision biopsy, *ER* estrogen receptor, *PR* progesterone receptor, *PPV* positive predictive value, *NPV* negative predictive value

### Correlation of CNB with surgical specimen for hormone receptor status in cases of neoadjuvant chemotherapy

The ER expression on CNB had a concordance rate of 100% (15/15 tumors) when compared to the surgical specimen.

The evaluation of PR expression showed a discordance in 12.5% (2/16 tumors) of CNB compared to the surgical specimen, with a switch from PR positive on CNB to PR negative in the surgical specimen after neoadjuvant chemotherapy. In both of those cases the ER and HER-2 receptor were concordant on CNB and surgical specimen.

### Correlation of CNB with surgical specimen for HER-2 receptor status in cases of neoadjuvant chemotherapy

When comparing the HER-2 expression, including FISH on CNB with the surgical specimen, there was a 4% discordance rate (1/25 tumors), due to one tumor being HER-2 positive in the surgical specimen, which previously was tested HER-2 negative.

### Number of CNB specimens as a marker of concordance/discrepancy

For 115 of 223 (51.6%) tumors the number of CNB’s was known. For the rest of tumors the amount of samples was summarized as “multiple”. The mean number of CNB’s was 2.96 with a standard deviation (SD) of 1.21. In the cases of receptor status discrepancy the amount of CNB’s was 2–3 for the cases of ER discrepancy (*q* = 0.569), 3–4 for PR discrepancy (*q* = 0.656) and 1–5 for HER2 discrepancy (*q* = 0.999). There was no significant difference in the amount of CNB’s taken when comparing the concordant and discrepant cases.

### Length of the CNB as a marker of concordance/discrepancy

The mean length of the histologic sample through CNB was 3.26 cm with a SD of 1.55 cm (min. 0.1 cm to max. 9.0 cm length). The mean length of the tissue sample of tumors with a hormone receptor discrepancy was 3.71 cm (SD of 1.35 cm) and there was no statistically significant association of the tissue sample with a concordant or discordant hormone receptor status (*q* = 0.313 for ER-Status and *q* = 0.777 for PR-status). Similar results were seen for the HER-2 discrepant tumors. HER-2 discrepant tumors had a mean length of tissue sample of 2.97 cm (SD of 1.26 cm), whereas HER-2 concordant tumors had a mean length of 3.27 cm (SD of 1.58 cm). Those differences were also deemed not significant (*q* = 0.999) for a prediction of concordance/discordance.

### Tumor size as an influencing factor of discrepancy

Most cases in this patient collective were T1 (45.7%) and T2 tumors (49.3%) with a mean tumor size of 2.36 cm. Tumors with an ER discrepancy (mean = 2.06 cm) were not significantly (*q* = 0.569) smaller than ER concordant tumors (mean = 2.37 cm). Similarly, statistically insignificant results were seen for cases of PR and HER-2 discrepancies, showing that tumor size is not a predictor for accuracy of CNB.

### Tumorgrading as an influencing factor of discrepancy

For 222 out of 223 (99.6%) tumors the histologic grading was known. Most tumors were G2 (149/222, 67.1%) and G3 tumors (52/222, 23.4%). Due to the small amount of G1 tumors with a discrepant receptor status, G1 and G2 tumors were pooled and compared to the group of G3 tumors. Discrepancies in ER-receptor status were found in seven tumors with G1/2 grading and 7 with a G3 grading, a result that did not show a statistical significance between the different gradings (*q* = 0.140). On the other hand, a PR-discrepancy was more common in tumors of G3 grading (8/45—17.8%) which was statistically significant. Regarding the HER2 status of tumors there was no significant differences of discrepancy between CNB and surgical specimen concerning the grading.

### Combined analysis of receptor concordance

To analyze the impact over all three receptor concordances (ER/PR/HER2) we utilized a multinominal linear regression (Table 5). Interestingly we could demonstrate a negative correlation with tumor grade (*p* = 0.05). Further, the amount of removed lymph nodes positively correlated with concordance (*p* = 0.04), while the amount of affected lymph nodes negatively impacted concordance rates (*p* = 0.02).

**Table 5:** Multinomial linear regression for the concordance and discrepancies between CNB and EB for all receptors (HER2/ER/PR)

Characteristics	Multinominal linear regression	
	*β*	*p*
CNB		
Cores	−0.07	0.79
Length/size	0.08	0.73
Breast surgery	−0.63	0.46
Invasive breast cancer		
Tumor size	0.95	0.18
Tumor grade	−1.37	0.05*
Axillary lymph node status	0.30	0.61
Histological tumor type	−0.14	0.67
Lymph nodes (affected)	−0.21	0.02*
Lymph nodes (removed)	0.14	0.04*

*CNB* core needle biopsy

*p ≤ 0.05, *β* change in odds of concordance

## Discussion

As one of the oldest and most widely used techniques for obtaining tissue, the sonographically guided CNB is currently a standard method for the diagnosis of unclear breast findings [2]. For patients with early breast cancer the sonographic evaluation of tumor size (T-stage) has recently been evaluated and confirmed as a highly accurate diagnostic method to determine the final pathologic size [3]. Continuous sonographic control of the needle position ensures precise and reliable sampling from palpable and non-palpable tumors. If the diagnosis of breast cancer is confirmed, histologic and biologic tumor characteristics are analyzed based on the CNB to initiate individually tailored adjuvant and neoadjuvant treatment.

A sufficient, representative amount of tissue with good tissue quality is necessary for the precise assessment of CNB; three to five biopsies are optimal [4, 5]. The use of small-caliber needles and the removal of only a few samples with a small amount of tissue can, in turn, reduce the sensitivity of the CNB [6]. A reliable diagnosis is usually made with the first biopsy, reaching a sensitivity of 96% with the second biopsy [7]. Based on an average of 3 CNBs, the CNB length was 3.26 cm (SD 1.55 cm). The differences in the number and size of CNBs were not statistically significant between concordant and discordant cases.

High agreement rates of 90.3–98.2% are reported for immunohistochemical detection of ER status [8–13]. The values for PR status are lower (77.9–89.3%) [11, 12]. Our results confirm the high concordance rate of 92.9% for ER status, but better results for PR concordance at 92.9%. ER was discrepant for 14 breast carcinomas (7.1%). These included 6 ER-negative breast carcinomas that were ER-positive in the CNB. The tendency of “up-scoring” the hormone receptor status is described in the literature [11, 14, 15]. Rapid fixation of the tissue samples means that more antigens are retained, which can be used to detect an immunohistochemically bound estrogen receptor [16–19]. A higher ER expression in CNB specimens can, therefore, possibly be explained by better fixation of the CNB compared to the surgical specimens. This may also mean that ER results based on the CNB more reliably identify those patients who will benefit from endocrine therapy [20]. The increased metabolic activity in the tumoral periphery can simulate increased ER expression, but this may not correspond to the actual expression in the tumor center [8, 16, 21].

Regarding HER2 status, there was a high degree of agreement between immunohistochemically HER2-negative (85.6%) and HER2-positive (78.6%) breast carcinomas. HER2 discrepancy occurred particularly in immunohistochemically HER2-unclear (score 2+) and HER2-negative breast cancers. In this regard, it is known that HER2 discrepancy is more often apparent in immunohistochemistry than in gene amplification analysis [22], although the [HercepTest™] with the modified scoring system has a high specificity of 93% [23]. HER2-positive tumor cells can be underrepresented by a CNB in a dominant HER2-negative breast carcinoma or vice versa [24]. The misinterpretation of a HER2-negative breast carcinoma with a concomitant HER2-positive DCIS cannot be ruled out either [25–27].

Additional FISH analyses offer a precise determination of the HER2 gene amplification. Compared to hormone receptors, the sensitivity (75.0%) and PPV (77.4%) for HER2 status were slightly reduced, while the specificity (96.2%) and NPV (95.7%) were reliable and accurate. If the recommended guidelines are followed, HER2 expression generally correlates with HER2 gene status [27, 28], although HER2 expression without gene amplification and gene amplification without HER2 expression have also been described [29]. In the context of polysomy of chromosome 17, the presence of ≥3 copies of the gene, in contrast to the typical single gene copy, may impede the differentiation between HER2 gene amplification and an increased gene number that is not associated with HER2 amplification [29, 30].

A more recent article by Slostad and colleagues that analyzed close to 1000 patients in a more recent time frame, has found similar concordance rates for the ER status and similarly higher discordance rates for PR and HER2 status. Nevertheless, they suggest that retesting for PR status should not be repeated due to a rarely clinically relevant change. Slostad and colleagues still raise the question of whether patients with HER2-equivocal results on CNB should be retested on the surgical specimen. Whether a repeated HER2 analysis of the surgical specimen could result in a survival benefit through added adjuvant systemic therapy unfortunately cannot be answered yet [31].

Another study by Rossi et al. analyzing the receptor status concordance of CNB with the surgical specimen in 923 patients has further analyzed a subgroup of ER-low-positive tumors which was found to have a higher rate of discrepancies. Furthermore, they found a high discrepancy rate for HER2-low tumors. Both factors were not analyzed in our study but further raise the question of whether confirmatory retesting should be performed in equivocal results [32].

Although the concordance rate of the CNB regarding the biomarker expression is high and similar to rates reported in the literature, the question has to be asked whether the discordance might be caused by intratumoral heterogeneity. The two mainly used hypotheses to describe the origin of intratumoral heterogeneity are the cancer stem cell hypothesis and the clonal evolution model [33]. The cancer stem cell model suggests that within a tumor, a cellular hierarchy exists, which sets a subset of so-called undifferentiated cancer stem cells (CSC) at the top tier with rapidly proliferating and terminally differentiated cells at the base. The principle of this theory is that this small CSC population is responsible for tumor growth, disease progression, and the generation of intratumoral heterogeneity [34]. The tenet of the clonal evolution model, on the other hand, is that cancer cells over time acquire different mutations and therefore different cellular characteristics, which in turn can confer a biologic advantage. Those advantages can result in a clonal expansion and therefore in a more profound intratumoral heterogeneity [35].

Interestingly, tumoral heterogeneity has been shown with regards to the receptor status comparing primary tumoral tissue with the receptor status of distant tumor cells (DTC) found in the bone marrow, therefore highlighting the importance of tumoral heterogeneity in an early breast cancer setting [36].

Whether or not the cases with a discordant receptor status are caused by intratumoral heterogeneity cannot be answered by this study.

Nevertheless, the accuracy of the CNB is similar to other studies [12, 31, 32, 37], but whether or not the CNB is accurate enough to cover the aspects of intratumoral heterogeneity cannot be answered with this study and remains to be elucidated. Nevertheless the accuracy of the CNB is similar to other studies [12, 31, 32, 37], but whether or not the CNB is accurate enough to cover the aspects of intratumoral heterogeneity cannot be answered with this study and remains to be elucidated.

### Limitations of the study

Due to the timing of data acquisition, the amount of neoadjuvantly treated patients is relatively low, whereas current guidelines prefer the use of neoadjuvant chemotherapy whenever systemic therapy is needed. This does not reflect the current standard of care, as the options of postneoadjuvant therapy options are increasing. Furthermore Ki67% was not routinely used in the years 2002–2005, while it is now a standard diagnostic parameter, which also guides therapy. For the same reason, cases were not classified as Her2-low or Her2-ultralow, as the concept was not of therapeutic significance within the timeframe covered in this study.

## Conclusion

CNBs were in good agreement with the surgical specimen in evaluating the receptor status of invasive breast cancer.

Thus, our findings further support the evidence that the sonographically guided CNB is an accurate way to diagnose the receptor status of breast cancer and support the recommendation that a sonographically guided CNB should be considered the initial diagnostic procedure to assess the receptor status of invasive breast cancer. Our study supports the recommendation of other studies, that confirmatory retesting of the surgical specimen in unequivocal results does not have to be performed.

## Data Availability

No datasets were generated or analysed during the current study.
